# Analysis of human capital effects introducing Bayesian quantile regression in the process of industrial structural upgrading

**DOI:** 10.1371/journal.pone.0304730

**Published:** 2024-07-08

**Authors:** Shaodong Shi, Xinbo Wang

**Affiliations:** Lomonosov Moscow State University, Moscow, Russian Federation; University of Almeria: Universidad de Almeria, SPAIN

## Abstract

In recent years, with the continuous evolution of the global economy and the adjustment of industrial structures, the understanding of the role played by human capital in the process of economic development has become particularly important. However, existing research on the impact of human capital on economic growth often adopts traditional regression methods, failing to comprehensively consider the heterogeneity and nonlinear relationships in the data. Therefore, to more accurately understand the influence of human capital on economic growth at different stages, this study employs Bayesian quantile regression method (BQRM). By incorporating BQRM, a better capture of the dynamic effects of human capital in the process of industrial structure upgrading is achieved, offering policymakers more targeted and effective policy recommendations to drive the economy towards a more sustainable direction. Additionally, the experiment also examines the impact of other key factors such as technological progress, capital investment, and labor market conditions on economic growth. These factors, combined with human capital, collectively promote the upgrading of industrial structure and the sustainable development of the economy. This study, by introducing BQRM, aims to fill the research gap regarding the impact of human capital on economic development during the industrial structural upgrading process. In the backdrop of the ongoing evolution of the global economy and adjustments in industrial structure, understanding the role of human capital in economic development becomes particularly crucial. To better comprehend the direct impact of human capital, the experiment collected macroeconomic data, including GDP, industrial structure, labor skills, and human capital, from different regions over the past 20 years. By establishing a dynamic panel data model, this study delves into the trends in the impact of human capital at various stages of industrial structure upgrading. The research findings indicate that during the high-speed growth phase, the contribution of human capital to GDP growth is 15.2% ± 2.1%, rising to 23.8% ± 3.4% during the period of industrial structure adjustment. Technological progress, capital investment, and labor market conditions also significantly influence economic growth at different stages. In terms of innovation improvement, this study pioneers the use of BQRM to gain a deeper understanding of the role of human capital in economic development, providing more targeted and effective policy recommendations. Ultimately, to promote sustainable economic development, the experiment proposes concrete and targeted policy recommendations, emphasizing government support in training and skill development. This study not only fills a research gap in the relevant field but also provides substantive references for decision-makers, driving the economy towards a more sustainable direction.

## 1. Introduction

The framework of this study is divided into five main sections. The first section is the introduction, which provides an overview of the research background and motivation, highlights the significance of the study, and outlines its objectives. The second section is recent research, which reviews the latest research relevant to the study’s topic, discusses key findings and perspectives from existing research, and identifies their limitations and unresolved issues. The third section is the research methodology, providing a detailed description of the Bayesian quantile regression method (BQRM) and data sources used in the study. It also introduces key variables, measurement indicators, and the research design. The fourth section presents the results and discussion, using charts, tables, and statistical data to showcase the study’s main findings. This section interprets these findings, relating them to the research questions and assumptions. The fifth section is the conclusion, which comprehensively summarizes the main outcomes of the study and addresses the research questions. The study also emphasizes its limitations and offers suggestions for future research directions. Lastly, the experiment underscores its importance and how it provides valuable insights for policymaking and practical applications in relevant fields.

With the continuous development of the global economy, many countries and regions consider industrial structural upgrading as a key strategic goal for achieving sustainable development. The transformation of industrial structure involves changes in the relative importance and contributions of various economic sectors, including the demand for labor skills, innovation dynamics, and the impact on international competitiveness [1–3]. For instance, according to international organization statistics, over the past decades, the share of manufacturing in many developed countries has declined, while the proportions of the service industry and high-tech sectors have gradually increased. This result indicates that the industrial structure is undergoing significant adjustments and reforms [4]. Furthermore, surveys targeting different industries and regions reveal that the application of emerging technologies is changing the production methods and business models of traditional industries, providing further evidence of the importance of industrial structural upgrading for economic development [5, 6]. The objective of this study is to address the research gap regarding the impact of human capital on economic development during the process of industrial structural upgrading, emphasizing its crucial role in driving sustainable economic growth. As the global economy continues to evolve and undergo industrial structural adjustments, understanding the role of human capital in economic development becomes particularly crucial. However, existing research often employs traditional regression methods when exploring this relationship, failing to comprehensively consider the heterogeneity and non-linear relationships in the data. Therefore, to more accurately comprehend the influence of human capital on economic growth at different stages, the experiment opts for BQR. This approach is better equipped to handle the heterogeneity and non-linear relationships in the data, providing more comprehensive and flexible posterior inferences. By introducing BQR, this study aims to more comprehensively capture the dynamic effects of human capital in the process of industrial structural upgrading. This not only contributes to filling the research gap in related fields but also offers policymakers more targeted and effective policy recommendations, steering the economy towards a more sustainable direction. Consequently, this study focuses on addressing the significant question of the direct impact of human capital on the economy and innovatively explores this relationship through BQR. Jedwab et al. [7] laid the theoretical groundwork for human capital accumulation and economic development, particularly focusing on the impact of work experience on wages and human capital accumulation. They provided theoretical foundations and empirical support for the relationship between human capital accumulation, returns to work experience, and economic development in this study. Gillman [8] proposed a model in which human capital deepens, education time increases, and labor transitions from agriculture. These significant trends in industrial development coincide with the gradual improvement in educational productivity levels. As education progresses, human capital becomes relatively abundant, and various sectors respond by deepening their investments in human capital. This study lays the groundwork for the conclusions that follow.

In the study of the impact of human capital on industrial structural upgrading, Aghaei et al. discusses the impact of green energy on economic growth. pointed out that human capital had a positive influence on economic growth, and the human capital index had a positive impact on the economic growth of the studied provinces [9]. Huang et al. examined the long-term effects of the human capital index, green energy, Economic Growth, and Gross Domestic Product (GDP) square on carbon emissions. They found that green energy and the human capital index also played a constructive role in reducing long-term and short-term environmental degradation [10]. Nouira et al. argued that export upgrading could only promote economic growth in countries that met certain prerequisites such as initial income levels, human capital, and institutional quality [11]. These studies provided valuable support for understanding the role of human capital in economic growth during the industrial structural upgrading stages discussed. Based on these findings, this study conducts an in-depth investigation into the role of human capital in industrial structural upgrading to reveal its importance for economic growth. In the process of reviewing existing literature, Jedwab provided the theoretical foundation for human capital accumulation, and Gillman contributed important theoretical insights, such as the model of deepening human capital, increasing education time, and transitioning from agriculture. Despite previous studies emphasizing the relationship between human capital and economic growth (such as research by Jedwab, Gillman, and others), there is still limited insight into its changing effects during different stages of industrial structural upgrading in current research. Therefore, the significance of this study not only lies in providing policymakers with insights into the complex relationship between human capital and industrial structural upgrading but also in filling gaps in existing research. Against this backdrop, the primary motivation of this study is to delve into the crucial role of human capital in industrial structural upgrading and the influence of multiple key factors, such as technological progress, capital investment, and labor market conditions, on this process. This study aims to understand under what circumstances human capital contributes most significantly to economic growth and how policies can be formulated to maximize the potential of human capital. In order to explore the relationship between human capital and economic development under the influence of multiple variables during the process of industrial structural upgrading, this study adopts BQR [12–14]. BQR is a non-parametric regression method based on Bayesian statistical principles. It handles heteroskedasticity and nonlinearity effectively, providing richer and more flexible posterior inference. Compared to traditional parameter regression methods like least squares or maximum likelihood, BQR can capture heterogeneity effects at different quantiles in the data, thereby providing more comprehensive and detailed information [15, 16].

The long-term relationship between national governance participation and national development in the member countries of the Turkish Council of Cooperation is a complex and crucial issue. National governance participation encompasses a range of indicators, including ’voice and accountability’ and ’government effectiveness,’ which are closely linked to national development. Keser et al. pointed out a significant causal relationship between these national governance indicators and the process of national development. These findings implied that, within the member countries of the Turkish Council of Cooperation, the government’s voice and accountability, as well as efficiency levels, play a crucial role in national development [17]. Specifically, government’s voice and accountability can enhance government transparency and accountability, thereby improving the quality of decision-making and implementation, fostering national development. Government effectiveness reflects the government’s capacity in policy implementation and provision of public services. An efficient government can more effectively drive the economic and social development of the country. Additionally, Özkök et al. discussed how fiscal federalism improves macroeconomic performance by enhancing the efficiency and performance of the public sector. While this study does not directly involve member countries of the Turkish Council of Cooperation, its results provide valuable reference [18]. Fiscal federalism can be seen as a form of national governance mechanism, enhancing government efficiency and performance through decentralization and collaboration, thereby promoting the economic development of the country. This study aligns with the findings of Keser et al., collectively emphasizing the crucial role of national governance in promoting national development. Therefore, the results of Keser et al. and Özkök et al.’s studies indicate that national governance participation has significant long-term impacts on the national development of the member countries of the Turkish Council of Cooperation. Government’s voice and accountability, as well as efficiency levels, are key factors determining the national development process, and governance mechanisms such as fiscal federalism can promote the macroeconomic performance of the government by improving efficiency and performance. These theoretical explanations contribute to a deeper understanding of the long-term relationship between national governance participation and national development in the member countries of the Turkish Council of Cooperation, providing crucial theoretical support for further research and policy formulation. This enables them to adopt targeted policies to promote the development of human capital and the upgrading of industrial structures based on the specific conditions of each region. In summary, Table 1 provides a clear insight into various provinces’ economic characteristics and industrial structures, offering robust support and a foundation for studying the relationship between human capital and industrial structural upgrading. The diversity and commonalities across these regions will provide a more comprehensive and in-depth perspective, enhancing the credibility and applicability of the research. Sultana et al. posited that various aspects of human capital positively impacted the growth of developing countries [19]. This study establishes a dynamic panel data model (PDM), capturing the changing trends of industrial structural development at various stages. It aligns with industrial organization theory, human capital theory, economic growth theory, regional economic theory, and technological progress and innovation theory. Technological progress, capital investment, and labor market conditions, are explanatory variables, while economic growth is the dependent variable.

**Table 1:** Comparison of different literature.

Classification	literature	Key Research Areas	Methods and Variables
Research related to industrial structure upgrading.	Zhang et al. (2019)	The impact of environmental regulations on industrial structural upgrading, focusing on the Beijing-Tianjin-Hebei region.	Empirical analysis considering environmental regulations and industrial structure.
	Jiang et al. (2020)	The influence of financial development and foreign direct investment on industrial structural upgrading.	Analyzing the impact of financial development, foreign direct investment, and industrial structure.
	Su et al. (2021)	The catalytic role of the digital economy in industrial structural upgrading, considering technological innovation.	Investigating the relationship between the digital economy, technological innovation, and industrial structure.
	Song et al. (2021)	The effects of environmental regulations on industrial structural upgrading, with a focus on strategic interactions among local governments.	Analyzing the influence of environmental regulations on local governments.
	Wang and Wang (2021)	A sustainable development perspective on industrial structural upgrading in China’s regions from the viewpoint of green finance.	Studying the relationship between green finance and industrial structure.
	Liu et al. (2022)	The relationship between the development of the digital economy, industrial structural upgrading, and green total factor productivity, considering Chinese cities.	Analyzing the interplay of the digital economy, industrial structure, and productivity.
	Wang et al. (2022)	Providing evidence for the impact of heterogeneous environmental regulations on industrial structural upgrading in China.	Researching the impact of different environmental regulations on industrial structure.
	Yin et al. (2024)	Industrial structural upgrading is regarded as a bridge between economic activities and the ecological environment, addressing environmental issues. It holds significant value in guiding the balance between environmental governance and economic development, as well as innovation-driven development strategies.	Analyzing the strategies of industrial structural upgrading for addressing environmental issues and its impact on economic development.
	Wang et al. (2024)	Exploring the relationship between resource dependency, the digital economy, and air pollution emphasizes enhancing air quality in resource-based cities by strengthening the digital economy.	Studying the relationship between resource dependency, the digital economy, and air pollution, and proposing recommendations for strengthening the digital economy.
	Liu et al. (2024)	By enhancing the impact of the environment on the economy through consumption upgrading, a consumption upgrading model has been constructed to examine the role of carbon emissions in high-quality economic development.	Analyzing the impact of consumption upgrading on economic development and researching the role of carbon emissions in economic development.
Current application status of BQR	Xu et al. (2019)	How industrial emissions affect healthcare expenditures in different regions.	Examining the relationship between industrial emissions and healthcare expenditures.
	Gayawan et al. (2019)	The spatial distribution of malnutrition among children under five years of age in Nigeria.	Analyzing the relationship between child malnutrition and geographical distribution.
	Alhamzawi and Ali (2020)	The R software package BRQ for Bayesian quantile regression.	Developing an R software package for Bayesian quantile regression.
	DinparastDjadid et al. (2021)	Modeling response times for automated system take over control.	Analyzing driver reaction times during the takeover of control from automated systems.
	Solaimani (2022)	The seasonal relationship between climate variables and evaporation in Iran’s southern Caspian Sea region.	Investigating seasonal relationships between climate variables and evaporation.
	Guan et al. (2023)	How spatial patterns of urban green space equity impact different equity levels.	Analyzing the relationship between urban green space equity and different levels of equity.
	Lu et al. (2024)	Exploring the heterogeneous effects of coordinated environmental regulatory policies on ecological resilience and the moderating role of industrial structure.	Analyzing the impact of coordinated environmental regulatory policies on ecological resilience and examining the moderating effect of industrial structure.

In summary, past research exploring the impact of human capital on economic development often relied on traditional regression methods, but these methods have certain limitations. They may not effectively handle the heterogeneity and non-linear relationships in the data, leading to an incomplete understanding of the relationship between human capital and economic development. Therefore, this study proposes BQR as a unique methodological approach in the study. Compared to traditional regression methods, BQR can more comprehensively capture complex relationships in the data, particularly excelling in dealing with potential heterogeneity and non-linear relationships. By adopting this method, the study can more accurately analyze the influence of human capital on economic growth at different stages, providing a new theoretical perspective and toolset. Thus, the theoretical contribution of this study lies in introducing a novel method to address the shortcomings of traditional research methods, bringing new insights and contributions to academic research in the relevant field.

## 2. Related work

### 2.1 Recent work on industrial structural upgrading

In recent years, industrial structural upgrading has emerged prominently in the field of economics, becoming a highly scrutinized and extensively researched subject. Numerous scholars are dedicated to exploring various factors influencing industrial structural upgrading, employing diverse research methods and perspectives. For instance, some studies indicate that the implementation of environmental regulatory policies has a significant impact on industrial structural upgrading [20]. Additionally, the driving forces of financial development and the digital economy have been found to be closely associated with industrial structural adjustments [21]. Furthermore, the development of emerging areas such as green finance is considered to be of significant importance for industrial structural upgrading [22]. Therefore, further research and evidence are needed to explore and substantiate the relationships between these factors and industrial structural upgrading. However, within the existing body of research, there has been a notable scarcity of attention directed towards the examination of the influence of human capital on industrial structural upgrading. In the study conducted by Zhang et al. (2019) [23], particular emphasis was placed on the examination of environmental regulations. The research involved a thorough investigation into the impacts of environmental regulatory measures on the process of industrial structural upgrading within the Beijing-Tianjin-Hebei region of China. Through empirical research, they observed a significant positive impact of environmental regulation on the upgrading of the overall industrial structure, as well as on the growth of green industries and high-end sectors. Their investigation utilized a PDM in conjunction with the difference-in-difference method to control for other potential influencing factors effectively. Similarly, Jiang et al. (2020) [24] delved into the intricate relationship between financial development and industrial structural upgrading in China. Furthermore, they explored the ramifications of outward foreign direct investment (OFDI) spillover effects on the process of industrial structural upgrading. The results showed that financial development positively promotes industrial structural upgrading, and the OFDI spillover effects further strengthen this promoting effect. The research methods included panel data regression models and Hausman tests. Su et al. (2021) [25] conducted an investigation into the potential promotion of industrial structural upgrading by the digital economy while also examining the mediating effect of heterogeneous technological innovation. The findings indicated a positive promoting effect of the digital economy on industrial structural upgrading, with the mediating effect exhibiting variations among different types of technological innovation. The research methods employed encompassed structural equation models and mediation effect tests. Similarly, Song et al. (2021) [26] explored the impact of environmental regulation on industrial structural upgrading by analyzing the strategic interactions of environmental regulation among local governments. Their results revealed a positive promotion of upgrading the overall industrial structure, green industries, and high-tech sectors due to environmental regulation. The study utilized the PDM and econometric methods for analysis. Furthermore, Wang and Wang (2021) [27] examined the impact of green finance on the upgrading of China’s regional industrial structure from the perspective of sustainable development. The research demonstrated that green finance played a significantly positive role in promoting industrial structural upgrading, particularly in regions characterized by energy and resource intensity. The research methods applied included econometric models and panel data analysis. Likewise, Liu et al. (2022) [28] investigated the relationship between the development of the digital economy, industrial structural upgrading, and green total factor productivity through empirical research on Chinese cities. The results indicated a positive correlation between the development of the digital economy and both industrial structural upgrading and green total factor productivity. In addition, Wang et al. (2022) [29] focused on studying the impact of environmental regulation on industrial structural upgrading from the perspective of its heterogeneity, conducting empirical analysis in China. Their research findings highlighted that different types of environmental regulation exert different effects on industrial structural upgrading. Lv (2021) [30] studied the security of IoT edge devices. The research pointed out that due to the large number and complex functionality of IoT edge devices, they faced security threats from network attacks and data leakage. In order to protect these devices, this study proposed a security scheme based on software practices and experience. Through experimentation, it was demonstrated that the scheme effectively protects IoT edge devices from the impact of security threats. Shi et al. (2022) provided crucial insights into the moderating effects of regional ownership structure on the relationship between value-added tax reform and industrial upgrading [31]. This study offered significant empirical support for a deeper understanding of the impact of value-added tax reform on regional industrial upgrading, emphasizing the pivotal role of regional ownership structure in this process. Wang and Shi et al. (2022) explored issues related to regional economic development under the backdrop of the “new normal.” They proposed an innovative method for identifying zombie enterprises and analyzed the characteristics of such enterprises in the Yangtze River Delta urban agglomeration during specific periods. The study presented recommendations to the government for establishing industrial policies [32]. The government should adjust its policy bias towards state-owned enterprises, considering regional economic characteristics to ensure the sustainable development of industries and regions. However, despite these valuable contributions, none of these studies systematically examines the specific impact of human capital in the process of industrial structural upgrading. Additionally, this study innovatively adopts BQR to systematically explore the changing trends of human capital in the process of industrial structural upgrading. Yin et al. (2024) pointed out that industrial structure upgrading, as a bridge between economic activities and the ecological environment, was one of the important strategies for addressing ecological environmental issues. Their findings had certain guiding significance and reference value for balancing environmental governance and economic development, effectively implementing innovation-driven development strategies, and formulating regional development strategies in China [33]. Wang et al. (2024) explored the correlation among resource dependency, digital economy, and air pollution, further investigating the mediating effects of income inequality and industrial upgrading. Empirical results called for a stronger emphasis on the digital economy in resource-based cities to improve air quality [34]. Liu et al. (2024) enhanced the impact of the environment on the economy through consumption upgrading, shifting the key driving factors of economic development from supply to demand. They constructed a consumption upgrading model to explore the role of carbon emissions in high-quality economic development [35]. The results showed that during the process of consumption upgrading, the marginal substitution rate of environmental demand by economic output demand exhibited a decreasing trend. Their study demonstrated that environmental resources could play roles in both supply and demand in economic output and consumer aspects, gradually becoming apparent during the process of consumption upgrading.

### 2.2 Recent work on BQR

In the current research on industrial structural upgrading, there has been relatively little exploration of the human capital effects. In order to address this research gap and comprehensively investigate the role of human capital in the process of industrial structural upgrading, the present study will incorporate the BQRM for analytical purposes. Xu et al. (2019) [36] employed the BQRM to study the impact of industrial exhaust emissions in different regions of China on healthcare expenditure. The research revealed regional variations in industrial exhaust emissions and their effects on healthcare expenditures. The findings provided a basis for the formulation of regional environmental protection policies and healthcare expenditure decisions. Gayawan et al. (2019) [37] used BQR to study the spatial distribution of malnutrition among children under five in Nigeria. The research identified significant regional disparities in malnutrition distribution, providing important evidence for targeted intervention measures and policy formulation. Alhamzawi et al. (2020) [38] introduced a software package named BQR, which provided researchers with a practical tool for analyzing data using BQRM applicable to various datasets and research questions. Alampi et al. (2021) [39] employed BQR to investigate the impact of prenatal exposure to toxic substances on autistic behaviors in children. The study found an association between prenatal exposure to toxic substances and autistic behaviors, offering new evidence for understanding the potential effects of toxic substances on child development. DinparastDjadid et al. (2021) [40] used BQR to establish a response time model for drivers regaining control from automated systems. The research revealed differences in drivers’ response times across different scenarios and individuals, providing guidance for designing and developing autonomous driving technologies. Solaimani (2022) [41] applied BQR to study the seasonal relationship between climate variables and evaporation in the southern region of Iran’s Lake Urmia. The research showed that seasonal climate changes significantly affected evaporation with varying degrees of impact, providing important references for water resource management in the region. Guan et al. (2023) [42] used BQR to study the spatial pattern impacts of urban green space equity under different levels of fairness. The research found spatial disparities in urban green space equity and different levels of fairness had varying effects on spatial patterns. The findings could provide decision support for urban planning and green space equity.

Human capital was previously regarded as a production factor in economic development but has gradually evolved into endogenous growth theory. Ozden & Guleryuz (2022) [43] employed machine learning techniques to investigate the relationship between economic development and human capital. This study used Bayesian-tuned support vector machines and Bayesian-tuned Gaussian process regression to optimize machine learning methods to establish an economic development forecasting model. Using different kernel functions, the Bayesian approach optimized hyperparameters, improving predictive performance. Dai et al. (2022) [44] pointed out that industrial structural upgrading implies a larger market size. Still, such upgrades often face significant challenges, such as human capital mismatches, directly impeding the vision of regional economic sustainability. Shen et al. (2023) [45] proposed that industrial and human capital structure upgrades are crucial for green development. With the increase in industrial structure intensity, the interactive effect of industrial structural upgrading and human capital structure upgrading on the efficiency of green development in resource-based cities shifted from positive to negative. Lu et al. (2024) employed BQR to explore the heterogeneous effects of coordinated environmental regulatory policies on ecological resilience from 2007 to 2021, and examined the moderating effect of industrial structure. The results indicated significant heterogeneity and variability in the impact of coordinated environmental regulatory policies on ecological resilience [46].

The BQRM has found wide applications across various fields. This study draws upon this method and applies it to explore the impact of human capital during the process of industrial structural upgrading. The application of this method aligns with research conducted by Gayawan et al. on the distribution of child malnutrition in Nigeria, the BQR software package BRQ developed by Alhamzawi et al., and the automated system takeover response time model established by DinparastDjadid et al. Additionally, the research by Ozden and Guleryuz reflects the changing trends of human capital evolving from an economic development factor to a vital component in the endogenous growth theory. Therefore, the BQRM employed in this study enables a more accurate capture of nonlinear effects, allowing for an in-depth investigation into the complex impact of human capital on industrial structural upgrading. Considering the dynamic nature of industrial structural upgrading, this study analyzes the influence of human capital at different stages, contributing to a more comprehensive understanding of the evolution of industrial structure. Ultimately, the results of this study assist decision-makers in better understanding how to optimize investments in human capital, promoting industrial structural upgrading.

### 2.3 Summary and analysis

The comparative analysis of these research references is presented in Table 1:

The BQRM has been widely applied in various fields. This study has demonstrated its superiority and flexibility in research related to environmental protection, health impacts, social equity, and transportation behavior. By summarizing and generalizing recent research on industrial structural upgrading, the connections between environmental regulation, financial development, the digital economy, and green finance with industrial structural upgrading have been extensively studied. Previous research has made significant contributions in the field of industrial structural upgrading. Firstly, a series of studies have explored the relationships between factors such as environmental regulations, financial development, the digital economy, and green finance with industrial structural upgrading, providing crucial references for the transition of industrial structures. For example, some studies suggest that the implementation of environmental regulations positively influences industrial structural upgrading, and the impetus from financial development and the digital economy is closely related to industrial structural adjustments. Moreover, research indicates that the development of emerging areas like green finance is of significant importance for industrial structural upgrading. Secondly, previous research has innovated in methodologies, employing various techniques such as PDM, structural equation models, and difference analysis for empirical analysis, providing technical support for in-depth studies on industrial structural upgrading. Additionally, some studies have focused on the moderating effects of regional ownership structure on the relationship between value-added tax reform and industrial upgrading, exploring the “new normal” in regional economic development and the issue of zombie enterprises, offering reference recommendations for government decision-making. Lastly, the role of human capital in industrial structural upgrading has not been thoroughly explored in previous studies. This study aims to fill this research gap by systematically investigating the role of human capital in industrial structural upgrading through BQRM. Unlike past studies that primarily focused on single influencing factors, this study comprehensively considers the impact of human capital on industrial structural upgrading, providing a new perspective and theoretical support to the field. The application of BQRM allows for a more accurate capture of the nonlinear effects of human capital on industrial structural upgrading, revealing its mechanisms and impact at different stages. This approach enables an in-depth exploration of the complex relationship between human capital and industrial structural upgrading, offering decision-makers more precise and practical policy recommendations. The innovation of this study lies in the systematic and comprehensive analysis of the impact of human capital on economic growth at different stages, introducing BQRM that provide richer and more flexible posterior inferences for addressing data heterogeneity and non-linear relationships, thus enhancing the understanding of the dynamic effects of human capital in the process of industrial structural upgrading. The application of this method offers a new perspective and approach to the study, helping to address methodological shortcomings in existing research. Furthermore, this study makes important contributions to policy recommendations. It emphasizes the importance of government support in training and skill development to promote the enhancement of human capital and sustainable economic development. These specific and targeted policy suggestions serve as a reference for the government in formulating relevant policies, contributing to steering the economy towards a more sustainable direction. Therefore, the contribution of this study lies in the comprehensive analysis of the impact of human capital on industrial structural upgrading through the introduction of BQRM, filling the research gap in existing literature, and providing new ideas and methods for research in this field. In the empirical discussion section, this study conducts a thorough analysis of the key factors influencing industrial structural upgrading. Firstly, prior research has primarily focused on individual influencing factors such as environmental regulations, financial development, or the digital economy, neglecting the intricate interactions among these factors. By comprehensively considering these factors, this study reveals the interrelationships among them and their combined impact on industrial structural upgrading, thus providing a new perspective for further expansion in the research field. Secondly, in the critical evaluation of the core research findings, this study employs a unified methodology and data source, enhancing the comparability of research results. This approach facilitates better comparison and validation with other research outcomes, thereby improving the credibility and academic value of the study. In this manner, the study effectively supplements and extends the limitations of previous research, offering a more comprehensive and in-depth understanding of the key influencing factors in the process of industrial structural upgrading.

In recent years, research on industrial structural upgrading has gained prominence in the field of economics, becoming a widely discussed and extensively studied topic. Many scholars have focused on exploring various factors influencing industrial structural upgrading, employing different research methods and perspectives. For example, factors such as environmental regulations, financial development, the digital economy, green finance, and IoT security have been extensively researched and closely linked to industrial structural upgrading [47–49]. None systematically investigated the specific impact of human capital in the process of industrial structural upgrading. Furthermore, the present study innovatively employs the BQRM to systematically explore the changing trends of human capital in the process of industrial structural upgrading. This method’s application is innovative, drawing inspiration from the approach used by Xu et al. (2019) in studying the impact of industrial emissions on healthcare expenditures across different regions in China. This study fills a research gap in the existing literature on the effects of human capital and employs a sophisticated analytical method to gain a deeper understanding of its dynamics. Moreover, the example of Guan et al. (2023) using BQR to study the equity of urban green spaces demonstrates the versatility of this method, which is capable of addressing spatial differences and providing support for decision-makers. The introduction of the BQRM in this study aligns with the growing trend in current research adopting this approach, as seen in studies like Gayawan et al. (2019) on malnutrition in Nigerian children under five and Alampi et al. (2021) on the impact of toxic exposure on autistic behaviors in children. Furthermore, the evolution of human capital from being viewed as an economic development factor to becoming an essential component in endogenous growth theory is reflected in Ozden and Guleryuz’s research (2022). This shift in understanding emphasizes the necessity of in-depth research into the impact of human capital in different economic contexts. In conclusion, while existing research has made significant strides in understanding industrial structural upgrading, this study provides a unique perspective by focusing on the often-overlooked aspect of human capital. The application of BQR offers a complex and in-depth analysis of the dynamic relationship between human capital and industrial structural upgrading, providing valuable insights for policymakers aiming to promote sustainable economic development.

This study utilizes BQR, a method that can more accurately capture nonlinear effects and delve into the complex impact of human capital on industrial structural upgrading. Furthermore, it considers the dynamism of industrial structural upgrading and examines the influence of human capital at different stages, contributing to a more comprehensive understanding of the evolution of industrial structure. Ultimately, the results of this study will assist policymakers in better comprehending how to optimize investments in human capital to facilitate industrial structural upgrading. In summary, this study supplements the limitations identified in the literature review by employing advanced methods and conducting a more comprehensive analysis. It also focuses on the dynamic nature of industrial structural upgrading. It is poised to provide policymakers with more specific and practical policy recommendations to promote sustainable industrial structural upgrading.

## 3. Establishing the analysis model of human capital effects using BQR and its relationship with structural upgrading

### 3.1 Analysis of the impact of human capital effects on economic growth

**Fig 1:**
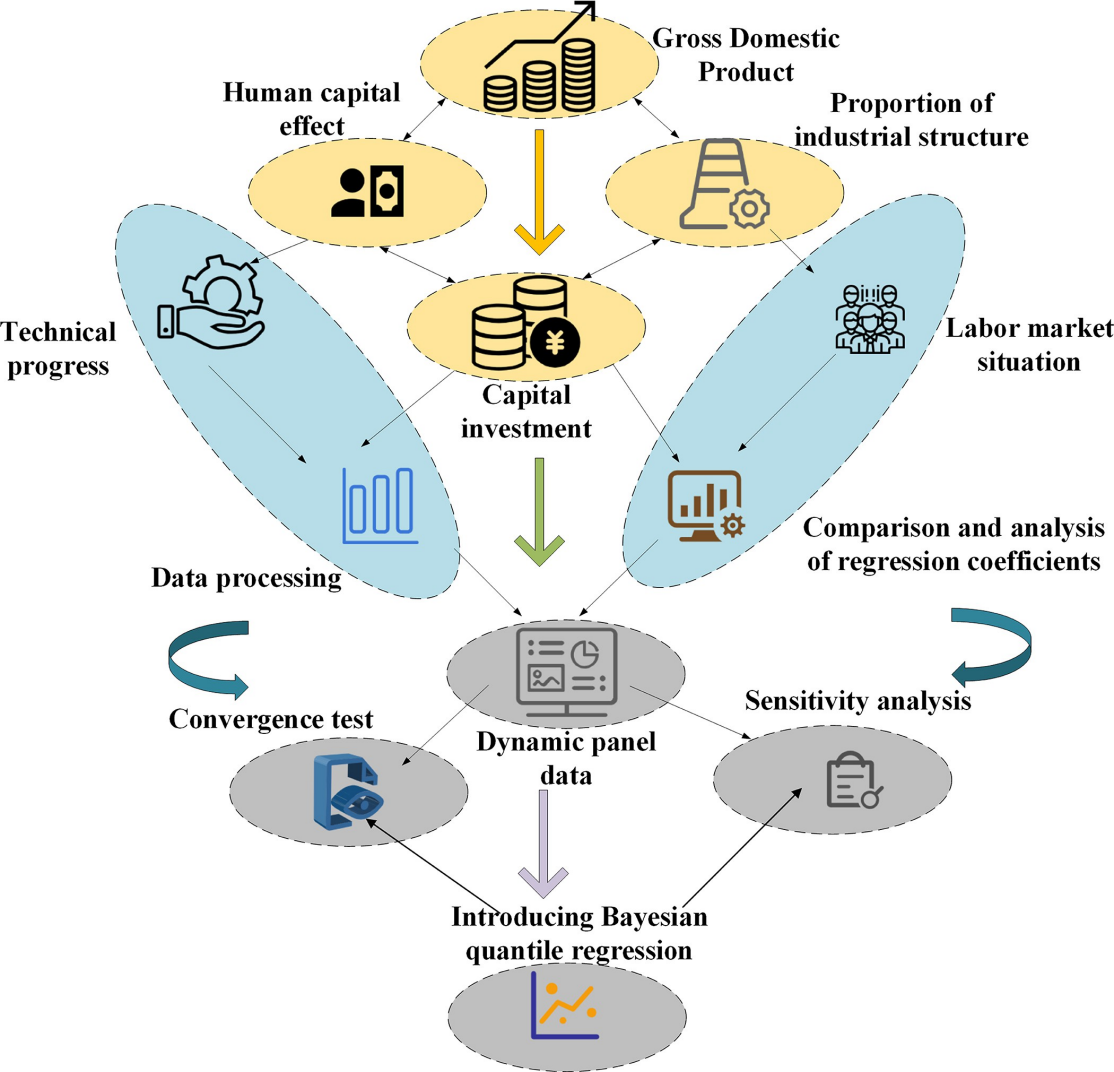
The impact of human capital effects on economic growth structure.

When exploring human capital’s impact on economic growth, it is crucial to control for double fixed effects to ensure the study’s accuracy and reliability. Double fixed effects refer to considering both time-fixed effects and individual fixed effects simultaneously in panel data analysis. Time-fixed effects primarily capture overall trends across time, while individual-fixed effects capture differences among individual entities, helping eliminate the influence of unobserved individual-specific fixed factors on the research results. In this study, GDP from different regions is initially used as a proxy variable for economic growth, with human capital indicator data serving as a proxy variable for human capital effects. Subsequently, the sample is divided into different groups based on the proportion of industrial structure, representing different stages of industrial structural development. This aids in more accurately observing the impact of human capital on economic growth at various stages. The focus of the study referenced in [50] lies in a systematic review of green human resource management in the service industry. The results indicate that organizations integrating environmental management with human resource management systems have achieved improvements at both organizational and employee levels. The research compares measurement criteria, theoretical frameworks, antecedents, outcomes, and mediator or moderator variables of green human resource management, proposing directions for future research. The study referenced in [51], using provincial-level panel data in China from 2014 to 2020, empirically analyzes the impact of digital economic development on China’s employment structure. The results show that the digital economy significantly influences China’s employment structure, exhibiting characteristics of both a “U-shaped” and “inverse U-shaped” curve. The study finds that digital economic development plays a certain role in promoting industrial structure and the stock of human capital. Therefore, in the era of the digital economy, supporting, promoting, and leading companies to accelerate digital transformation, enhancing employee knowledge and skills, and unleashing the dividends of human capital are crucial. The research in [52] explores the relationship between human capital, urbanization, and ecological footprint. The study reveals that globally, human capital initially increases and then decreases the ecological footprint. Urbanization plays a linear moderating role in the impact of human capital on the ecological footprint. Therefore, different countries should continue to enhance human capital, promote industrial structure upgrading, green technological innovation, and changes in energy-efficient lifestyles. Next, a dynamic PDM is adopted to capture the influence of human capital effects on economic growth during different stages of industrial structural development while controlling for other explanatory variables such as technological progress, capital investment, and labor market conditions. Additionally, it can comprehensively analyze the impact of human capital on different industrial structure development stages. Lastly, the BQRM is employed to estimate the model parameters and conduct a comparative analysis of the regression coefficients across various quantiles, thereby elucidating the heterogeneity and nonlinearity of the influence of human capital effects on economic growth. This step helps reveal the heterogeneity and nonlinear characteristics of the impact of human capital effects on economic growth at different quantiles. By introducing double fixed effects, the study better controls for time and individual fixed effects, enhancing the endogeneity and interpretability of the research. The outcomes pertaining to the effects of human capital on economic growth are illustrated in Fig 1:

In addition to human capital, technological progress, capital investment, and labor market conditions have also been confirmed to have significant impacts on industrial structural upgrading and economic growth. Technological progress provides impetus for industrial structural upgrading by enhancing productivity and innovation capabilities. Capital investment, as a source of funds for enterprise expansion and technological renewal, plays a crucial role in accelerating the optimization and upgrading of industrial structure. Labor market conditions, including the balance of labor supply and demand, educational levels, and skills training, directly affect the allocation and utilization efficiency of human capital in the process of industrial structural upgrading. The interaction and synergistic effects of these factors collectively drive high-quality economic development.

### 3.2 Application of BQRM in the study of human capital effects

In order to analyze the human capital effects using the BQRM, this study first needs to determine the form and parameters of the model [53, 54]. The following BQRM is adopted, as shown in Eq (1):

$$y_{it}=\alpha_{i}+\beta_{\tau }x_{it}+\gamma_{\tau }z_{it}+\epsilon_{it}$$ (1)

In Eq (1), *y*_*it*_ represents the economic growth rate of the *i*-th region in the *t*-th time period. *x*_*it*_ represents the human capital indicator of the *i*-th region in the *t*-th time period. *z*_*it*_ represents other explanatory variables of the *i*-th region in the *t*-th time period (such as technological progress, capital investment, labor market conditions, etc.). *α*_*i*_ represents the fixed effect of the *i*-th region, *β*_*τ*_ and *γ*_*τ*_ represent the regression coefficients at different quantiles τ. *ϵ*_*it*_ represents the error term. Next, this study needs to specify the prior and posterior distributions of the model parameters. This study assumes that the model parameters follow a normal distribution, as shown in Eqs (2)–(4):

$$\alpha_{i}∼N(\mu_{\alpha^{\prime }},\sigma_{\alpha }^{2})$$ (2)

$$\beta_{\tau }∼N(\mu_{\beta },\sigma_{\beta }^{2})$$ (3)

$$\gamma_{\tau }∼N(\mu_{\gamma },\sigma_{\gamma }^{2})$$ (4)

In Eqs (2)–(4), *μ*_*α*′_ and $\sigma_{\alpha }^{2}$ represent the mean and variance, respectively. This investigation chooses suitable prior distribution parameters based on data characteristics and prior knowledge. Subsequently, the Markov Chain Monte Carlo (MCMC) method generates the posterior distribution of model parameters and conducts convergence tests and sensitivity analysis.

**Fig 2:**
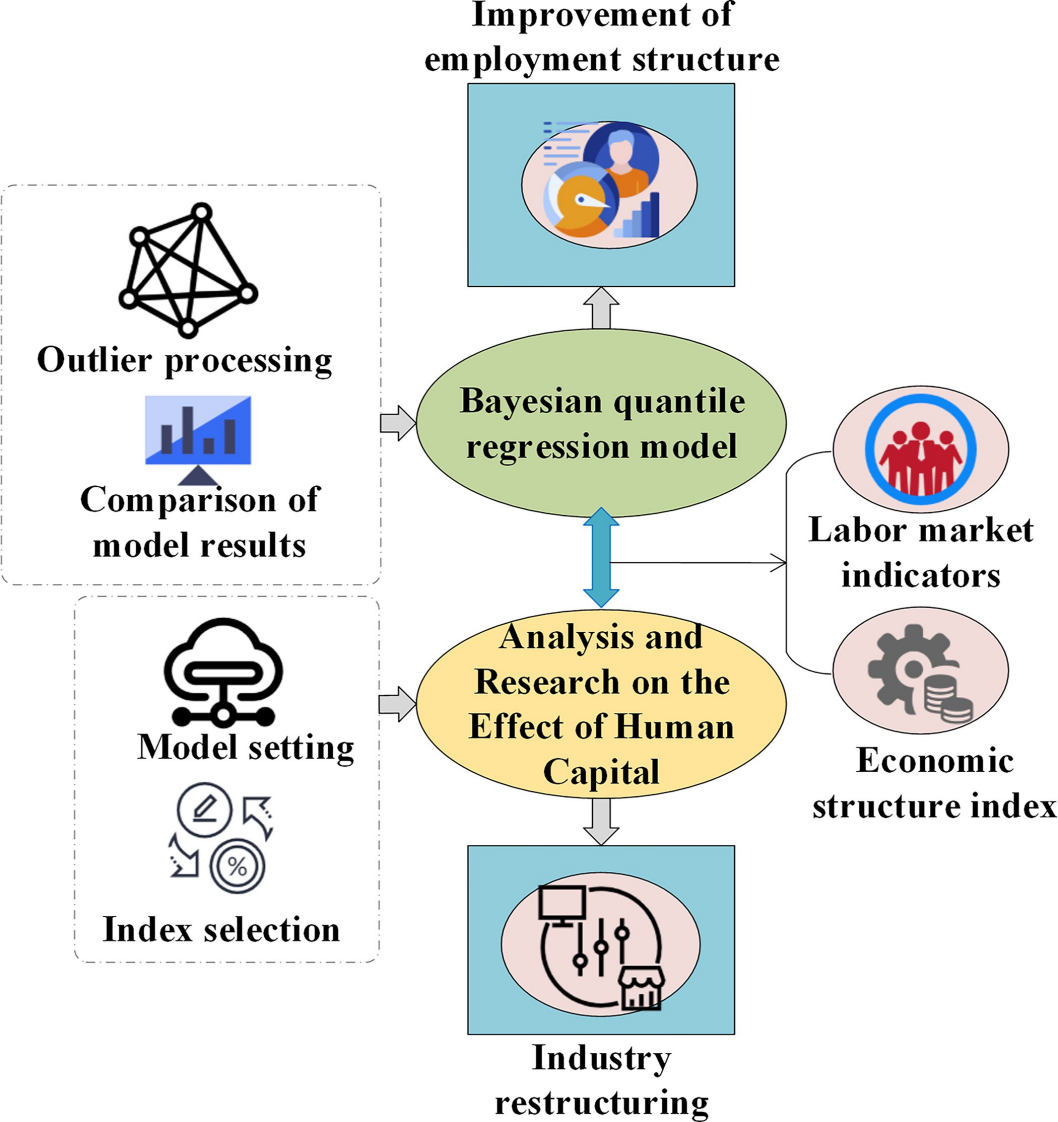
Structure of the application of BQRM in the study of human capital effects.

Finally, based on the posterior distribution of model parameters, this study estimates the impact of human capital effects on economic growth at different quantiles. The structured process of applying the BQRM in the examination of human capital effects is illustrated in Fig 2:

### 3.3 Data collection and sample construction method

The study selected data from the past 20 years (2001–2020), covering 31 provincial-level administrative regions in China. In this study, each region’s real GDP was employed as a surrogate variable for economic growth, and the relevant data were obtained from the National Bureau of Statistics and the World Bank. As for the industrial structure, the proportions of the primary, secondary, and tertiary industries in the GDP of each region were utilized as proxy variables, with data sourced from the National Bureau of Statistics and the World Bank. For human capital, average years of education, the number of higher education graduates, and the number of scientific and technological personnel in each region were used as proxy variables, with data sourced from the China Education Statistics Yearbook and UNESCO. After data collection, the study conducted data cleaning, processing, and standardization to address outliers, missing values, and dimensional differences. Then, based on the proportion of industrial structure, the samples were divided into different groups representing different stages of industrial structural development. The relevant description statistics of the data collection and sample construction methods are presented in Table 2:

**Table 2:** Description of statistics of data collection and sample construction methods.

Type of data	Data processing	Sample division
GDP	Logarithmic and Percentage Transformations	Cluster Analysis or Factor Analysis
industrial structure	Logarithmic and Percentage Transformations	Cluster Analysis or Factor Analysis
human capital	normalize or normalize	Human Capital Index or Human Capital Quality
skill improved	differential or growth rate	Technological Progress Intensity or Technological Progress Efficiency
capital investment	Missing value imputation or outlier removal	capital investment elasticity or labor market elasticity
labor market	Missing value imputation or outlier removal	capital investment elasticity or labor market elasticity

This study collected macroeconomic data, including GDP, the composition of industrial sectors, labor force skills, and human capital indicators, from various regions over the past two decades. The data encompassed 31 provinces in China, excluding Hong Kong, Macau, and Taiwan. Table 3 presents some of these provinces’ economic characteristics and distribution of industrial structures.

**Table 3:** Economic characteristics and distribution of industrial structures in different provinces.

Number	Province	Economic characteristics	Industrial structure distribution (proportion of GDP)
1	Beijing	The region’s main economic characteristics are finance, technology, and real estate, with the service industry dominating the area.	Tertiary industry (80%), primary industry (<1%)
2	Shanghai	The region’s main economic characteristics are finance, shipping, and manufacturing, with developed service and manufacturing industries.	Tertiary industry (70%), Secondary industry (29%)
3	Guangdong	The province’s main economic characteristics are manufacturing, electronics, and export trade, with manufacturing taking the lead.	Secondary industry (53%), Tertiary industry (45%)
4	Zhejiang	The province’s main economic characteristics are manufacturing, electronics, and finance, with equal emphasis on manufacturing and service industries.	Secondary industry (48%), Tertiary industry (50%)
5	Jiangsu	The province’s main economic characteristics are manufacturing, electronics, and chemicals, with developed manufacturing and service industries.	Secondary industry (49%), Tertiary industry (48%)
6	Henan	The province’s main economic characteristics are agriculture, manufacturing, and construction, with agriculture still playing an important role.	Primary industry (30%), secondary industry (40%)
7	Sichuan	The province is characterized by energy, agriculture, and manufacturing as its main economic features, with agriculture and manufacturing dominating.	Primary industry (27%), secondary industry (45%)
8	Hubei	The province’s main economic characteristics are manufacturing, biomedicine, and automobiles, with developed manufacturing and service industries.	Secondary industry (49%), tertiary industry (48%)
9	Shandong	The province’s main economic characteristics are manufacturing, electricity, and agriculture, with agriculture and manufacturing dominating.	Primary industry (27%), secondary industry (48%)
10	Shannxi	The province’s main economic characteristics are energy, manufacturing, and metallurgy, with developed manufacturing and mining industries.	Secondary industry (49%), tertiary industry (48%)
11	Hunan	The province’s main economic characteristics are agriculture, manufacturing, and rural finance, with agriculture still playing an important role.	Primary industry (27%), secondary industry (48%)
12	Yunnan	The province’s main economic characteristics are agriculture, mining, and tourism, with agriculture and mining dominating the economy.	Primary industry (31%), secondary industry (47%)
13	Shanxi	The province is characterized by energy, metallurgy, and manufacturing as its main economic characteristics, with energy and manufacturing taking the lead.	Primary industry (30%), secondary industry (48%)
14	Liaoning	The province’s main economic characteristics are heavy industry, energy, and manufacturing, with developed manufacturing and mining industries.	Primary industry (29%), secondary industry (51%)
15	Anhui	The province’s main economic characteristics are manufacturing, agriculture, and construction, with agriculture and manufacturing dominating.	Primary industry (28%), secondary industry (44%)

Table 3 displays the economic characteristics and industrial structures of various provinces in China, reflecting the diversity in each province’s economic features and industrial layouts. This data provides crucial background information for investigating the impact of human capital on industrial structural upgrading. The economic characteristics and distribution of industrial structures across different provinces highlight distinct regional economic development patterns. Such regional variations offer diversity for the study, aiding a more comprehensive understanding of the role of human capital in varying economic contexts. The main economic features and distribution of industrial structures in each province form the basis for understanding changes in industrial structures. By comparing different provinces, it becomes possible to identify the impact of human capital on economic development under different industrial structures, providing references and benchmarks for the study. The primary economic features of each province also reflect the regional emphasis on industrial development, offering guidance to policymakers.

Descriptive statistical results of the data in Table 4: The average overall economic level during the study period is relatively high, reflecting the overall robust development of the economy. Secondly, regarding industrial structure, the average industrial structure is 32.45%, indicating the average distribution of industrial structures across regions. However, there is a significant variance in industrial structure, with a standard deviation of 3.51, suggesting considerable differences in industrial structures across regions, possibly influenced by regional economic characteristics and policies. The average level of human capital is relatively high, indicating that regions, on the whole, have achieved certain accomplishments in cultivating and developing human capital. Additionally, the variation in human capital is relatively small, reflecting a more balanced level of talent development. Concerning skill enhancement, the average skill enhancement is 8.56%, reflecting the average growth rate of skill levels across regions. However, there is significant variance in skill enhancement, implying notable differences in skill development across regions, necessitating in-depth research into the underlying reasons and influencing factors. Regarding equipment investment and the labor market, there are significant differences in both the average levels and variances across regions. This suggests that different regions exhibit notable disparities in equipment investment and labor market conditions, likely associated with factors such as local policies, industrial structure, and economic foundations.

**Table 4:** Descriptive statistical results of the data.

Type of data	Mean	Variance	Standard deviation	Unit of measurement
GDP	125.67	45.89	6.77	Billion
Industrial structure	32.45	12.34	3.51	Percentage
Human capital	0.78	0.09	0.30	Standardized score
Skill improvement	8.56	1.23	1.11	Percentage growth rate
Equipment investment	58.34	17.45	4.18	Billion
Labor market	22.67	9.56	3.09	Percentage

This study has thoroughly taken into account the latest research findings in formulating research questions and hypotheses. Aghaei et al. underscored the pivotal impact of human capital on industrial structural upgrading. Huang et al. revealed the close connection between human capital and economic growth. In light of these recent research discoveries, the study puts forward the following research questions and hypotheses: The relevant assumptions established are shown in Table 5. BQR is employed to conduct an in-depth analysis of the effects of industrial structural upgrading on human capital. This study’s contribution lies in applying BQR to the study of industrial structural upgrading and its impact on human capital, providing a novel analytical tool and perspective in this field. Moreover, meaningful and insightful conclusions and policy recommendations are drawn, which can serve as references for promoting industrial structural upgrading and human capital development.

**Table 5:** Research hypothesis.

Number	Research hypothesis	Description	The basis of the literature
Hypothesis 1	There is a positive correlation between human capital and economic growth.	Human capital refers to the portion of a country or region’s population possessing high-quality education and skills. The experiment hypothesizes that with an increase in the level of human capital, labor productivity will rise, thereby driving economic growth. This could be attributed to the fact that well-educated and trained labor tends to be more creative and productive, effectively utilizing existing resources.	Taking the research by Sultana et al. as an example, they found that the enhancement of human capital has a significant positive impact on the economic growth of developing countries. Their study suggests that by elevating the level of human capital, a nation can bolster the productivity and creativity of its workforce, thereby fostering economic growth.
Hypothesis 2	The impact of industrial structural upgrading on human capital is dynamic.	In this hypothesis, industrial structural upgrading is the independent variable, while human capital is the dependent variable. This hypothesis suggests that the impact of industrial structural upgrading on human capital is multi-stage and dynamic. In the early stages, there might be a shortage of skills or unemployment situations. However, with economic development and industrial structural upgrading, the demand for and value of human capital may gradually increase.	Relevant studies indicate that industrial structural upgrading may lead to changes in the labor market, influencing the demand and value of human capital. For instance, research by Bye et al. revealed that the transformation of industrial structure can increase the demand for certain industries, consequently raising the value of related skills and promoting the development of human capital [55].
Hypothesis 3	Technological progress, capital investment, and labor market conditions significantly affect economic growth.	The experiment hypothesizes that factors such as technological progress, capital investment, and labor market conditions play a crucial role in economic growth. Technological progress can enhance productivity, capital investment can increase production capacity, and labor market conditions directly affect labor supply and efficiency.	Drawing on related research, Bongers et al. pointed out that technological progress is a key factor driving economic growth [56]. Their study demonstrates that technological advancement can enhance productivity and efficiency, thereby facilitating long-term economic growth. Additionally, Ogbeifun’s research indicated that capital investment also has a significant positive impact on economic growth, as it can increase production capacity and drive economic development [57].
Hypothesis 4	The introduction of BQRM allows for a more in-depth analysis of the dynamic and phase-specific characteristics of the impact of human capital on economic growth.	At different stages of industrial structural upgrading, the contribution of human capital to economic growth varies. The introduction of BQRM helps to reveal this dynamic change, thus providing policymakers with more targeted policy recommendations.	Although BQRM are less commonly used in economic research, some recent studies suggest that they perform better in capturing nonlinear relationships and extreme values. For example, research by McGough et al. indicated that Bayesian methods exhibit higher flexibility and accuracy when dealing with complex datasets [58].

### 3.4 Analysis of the moderating effect of structural upgrading on human capital effects

In order to compare the accuracy and precision of the BQRM with the Ordinary Least Squares Method (OLSM) and PDM in analyzing the impact of human capital on industrial structure models [59, 60], Ma et al. (2023) examined the relationship between advanced human capital structure, industrial intelligence, and the structure of the service industry. The results indicated that an advanced human capital structure significantly promoted the development of the service industry. Additionally, there is a certain heterogeneity in the impact of an advanced human capital structure on the service industry [61]. This study will undertake a multifaceted evaluation. This evaluation will encompass various aspects, including the contribution rate of human capital, technological progress, capital investment, labor market conditions, industrial structure adjustment index, and employment structure improvement index. The human capital contribution rate is employed to quantify the contribution of an advanced human capital structure to the development of the service industry. Considering the importance of technological progress in economic development, technological progress will be assessed for its impact on the development of the service industry structure, as mentioned by Ma et al. (2023). Given the crucial role of capital investment in industrial structural upgrading, this study examines the impact of capital investment on the service industry structure. Understanding the state of the labor market is crucial for comprehending the development of the service industry structure. This study will assess the relationship between the labor market conditions and the upgrading of the service industry structure. The industrial structural adjustment index will help understand the overall adjustment of the industrial structure and, consequently, evaluate the contribution of an advanced human capital structure to industrial structural upgrading. By examining the improvement in employment structure, the study can assess the impact of an advanced human capital structure on employment structure, providing a more comprehensive understanding of its socio-economic effects.

In order to enhance the reliability of the assessment, two sets of data, labeled BQRM1 and BQRM2, will be applied to the BQRMseparately in the experimental analysis. In the experimental analysis, this study will divide the period from 2001 to 2020 into five distinct stages (each spanning four years), denoted as A, B, C, D, and E. Specifically, stages A, B, and C represent periods of high-speed industrial growth, while stages D and E represent periods of industrial structural adjustment. By focusing on the impact of rapid industrial growth and industrial structural adjustments at different stages, the study can provide a more specific and refined understanding of the evolving process of the Chinese economy during this period. Stage A: The years 2001 to 2004 represent a period of rapid industrial growth. During this stage, the economy underwent swift industrial expansion to meet the prevailing economic demands. Stage B: Spanning from 2005 to 2008 still signifies a period of rapid industrial growth. This continued growth is attributed to various factors such as technological innovation, market demand, leading to a rapid increase in industrial output. Stage C: Encompassing the years 2009 to 2012, it continues to characterize a period of rapid industrial growth. This phase witnessed a series of factors promoting industrial expansion. Stage D: Covering the years 2013 to 2016 represents a period of industrial structural adjustment. This is a phase of economic adaptation and optimization, where businesses and industrial structures may have undergone adjustments to adapt to new market and policy environments. Stage E: Spanning from 2017 to 2020, continues to represent a period of industrial structural adjustment. This is a phase of deeper adjustments to cope with global economic and industrial changes. The specific analytical process is outlined in Table 6:

**Table 6:** Analysis of the moderating effect of structural upgrading on human capital effects in industrial structure upgrading.

Influencing factors	industrial restructuring	human capital effect
Technological progress and innovation	Traditional industries declined, and emerging industries rise	Improving human capital and promoting innovation
Education and training	Changes in demand lead to workforce restructuring	Education and training improve workforce skills
Labor market demand and supply	Changes in labor demand and industry matching	Market fit affects industrial efficiency
policies and institutions	Industrial policy and institutional reform	Policies affect talent allocation
Cross-border investment and trade liberalization	Foreign capital inflows and changes in industrial structure	International competition promotes the optimal allocation of talents
Differences in urban and rural development	Urbanization promotes the industrial agglomeration effect	Rural education and training impact human resources

## 4. Results

The study selects OLSM, PDM, and BQRM as the main analytical methods. The choice of these methods is based on their significant advantages in addressing the research questions and their ability to provide more reliable and interpretable results. First, OLSM is widely used in economic research due to its simplicity, ease of computation, and good fit for linear relationships. It is suitable for analyzing the relationship between industrial structure and economic growth in the research, providing preliminary analysis results and insights into the impact trends of different industrial structure changes on economic growth. Second, the PDM has certain advantages in dealing with panel data. The PDM is better at controlling the impact of individual characteristics and time variation on data, thereby improving the accuracy and robustness of the model. For the study with a long time span and multidimensional data, the PDM can more comprehensively capture the impact of industrial structural changes on economic growth. Finally, the BQRM, as a Bayesian statistical method, performs well in handling nonlinear and heteroscedastic data. It offers a more flexible modeling approach and better addresses the complexity of real-world data. The BQRM can more accurately capture the nonlinear impact of industrial structural changes on economic growth. In addition to these main methods, the study includes an analysis of dynamic panel data to more comprehensively consider the characteristics of time series and panel data. Simultaneously, the experiment examines the cross-sectional analysis results to ensure the research methods and data analysis are comprehensive and reliable. The integrated application of these analytical methods provides a more comprehensive and accurate understanding of the relationship between industrial structure and economic growth, offering more reliable and convincing conclusions for the research.

### 4.1 Analysis of human capital and technological progress performance

**Fig 3:**
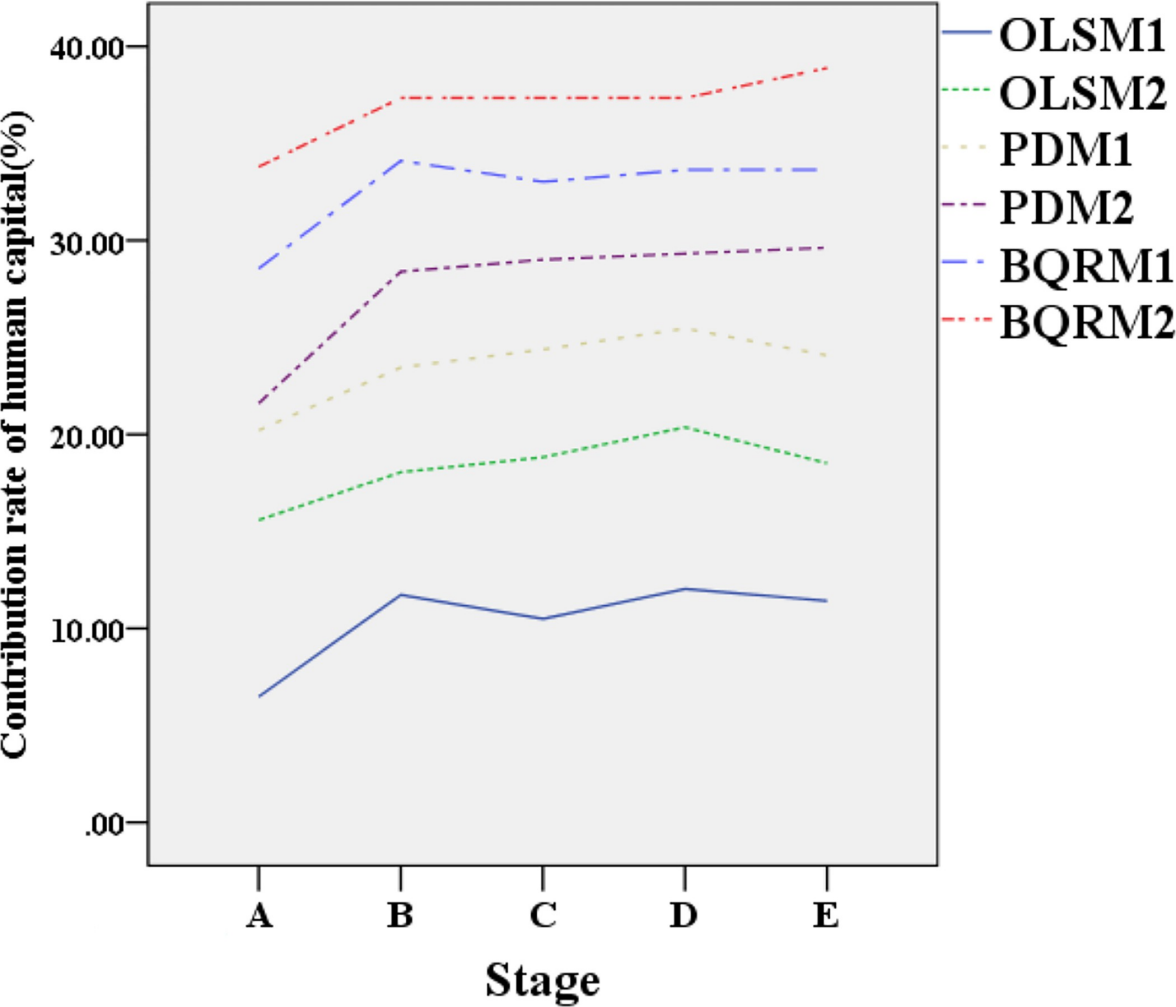
Data variation curves of human capital contribution rates in different industrial structure models at different time points.

**Fig 4:**
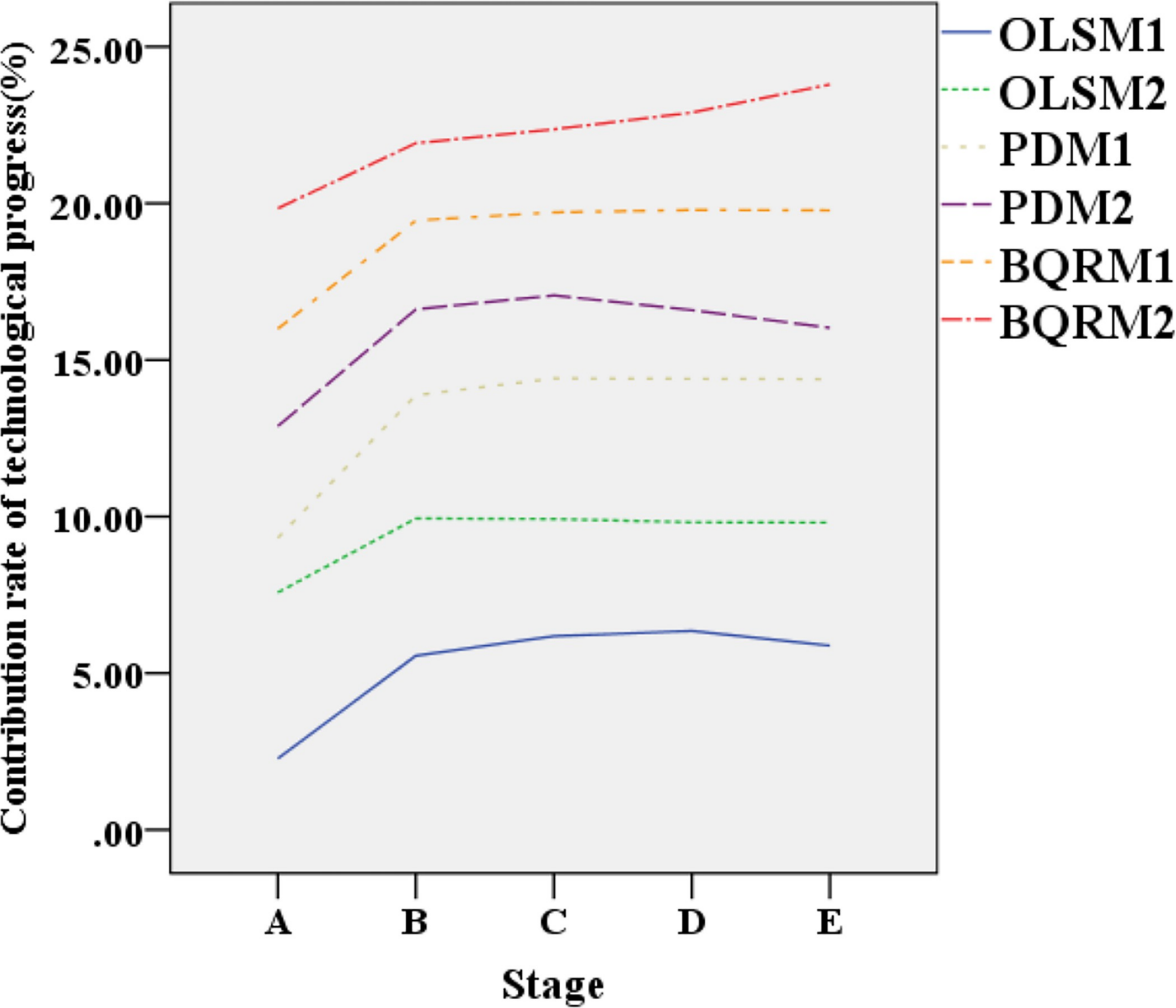
Data variation curves of technological progress contribution rates in different industrial structure models at different time points.

Fig 3 displays the data variation curves of human capital contribution rates in different industrial structure models at different time points. Fig 4 presents the data variation curves of technological progress contribution rates in different industrial structure models at different time points.

Fig 3 provides a detailed analysis of the contribution rates of human capital and technological progress for different industrial structure models at various time points. During different stages of economic development, each model exhibits varying trends in the contribution rates of human capital and technological progress. Taking human capital as an example, during the high-speed growth phase, its contribution rate to GDP is approximately 15.2% ± 2.1%, while during the period of industrial restructuring, this contribution rate further increases to 23.8% ± 3.4%. OLSM2, PDM2, and BQRM2 show higher contribution rates of human capital at each time point, indicating that these models have a significant impact on current or future industrial development. Additionally, with the passage of time, the growth rate of the contribution rate of human capital gradually slows down, revealing a trend towards the stabilization of the contribution of human capital to industrial development. These findings highlight the importance of human capital to economic growth and its operating mechanism at different stages of development.

In Fig 4, the technological progress contribution rates of different industrial structure models show diverse trends at different time points. This result indicates that each model contributes to technological progress at different stages. Overall, the technological progress contribution rates show an increasing trend at different time points, suggesting that technological progress’s driving effect on industrial development gradually strengthens. In the early stages A and B, each model’s technological progress contribution rates are relatively low but gradually increase over time. This may be because technological innovation requires some time for accumulation and dissemination before it can fully unleash its potential. In the subsequent stages C, D, and E, the technological progress contribution rates of different models remain relatively stable.

### 4.2 Comparison of capital investment and labor market contribution rates

**Fig 5:**
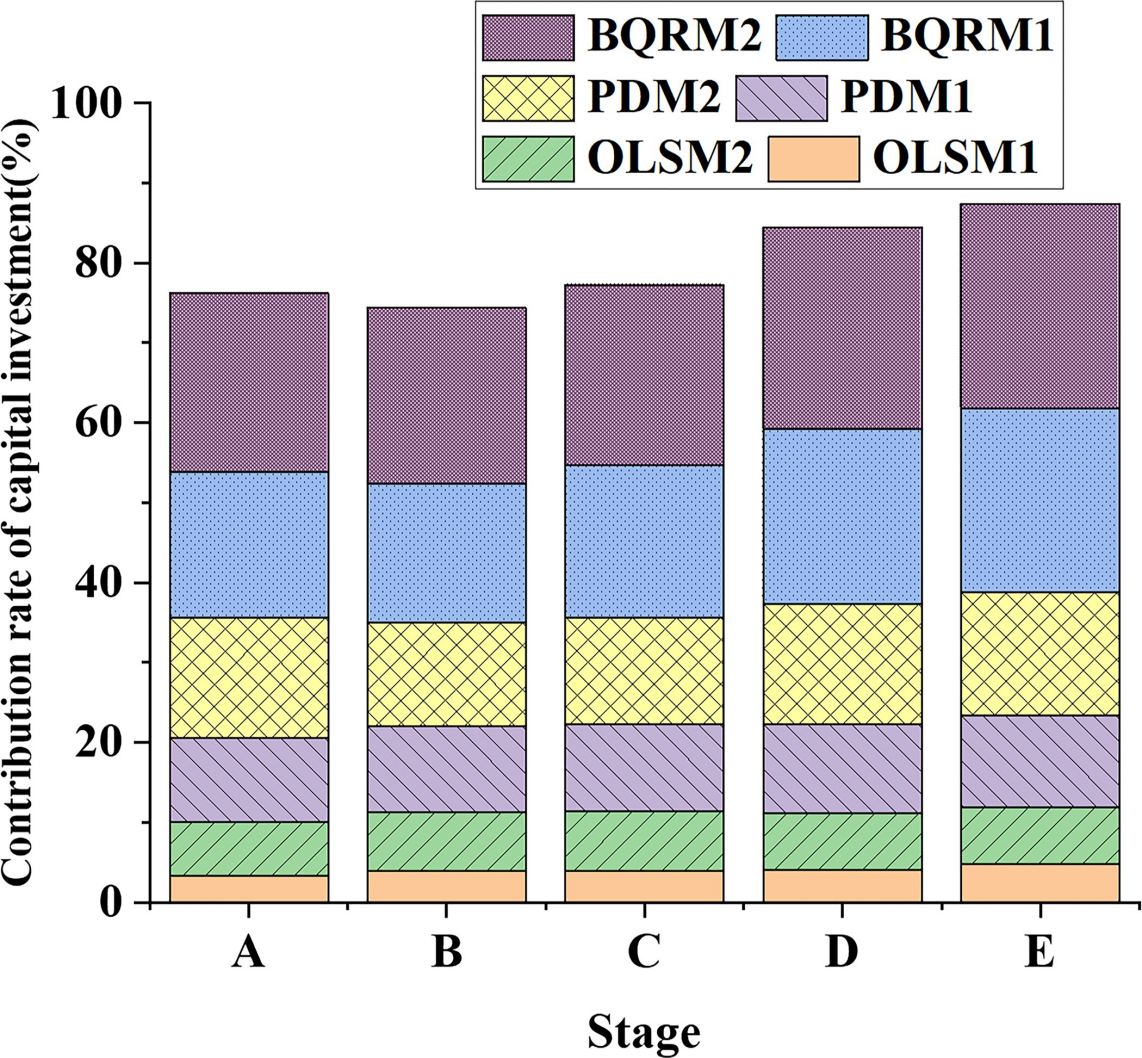
Data variation curves of capital investment contribution rates in different industrial structure models at different time points.

**Fig 6:**
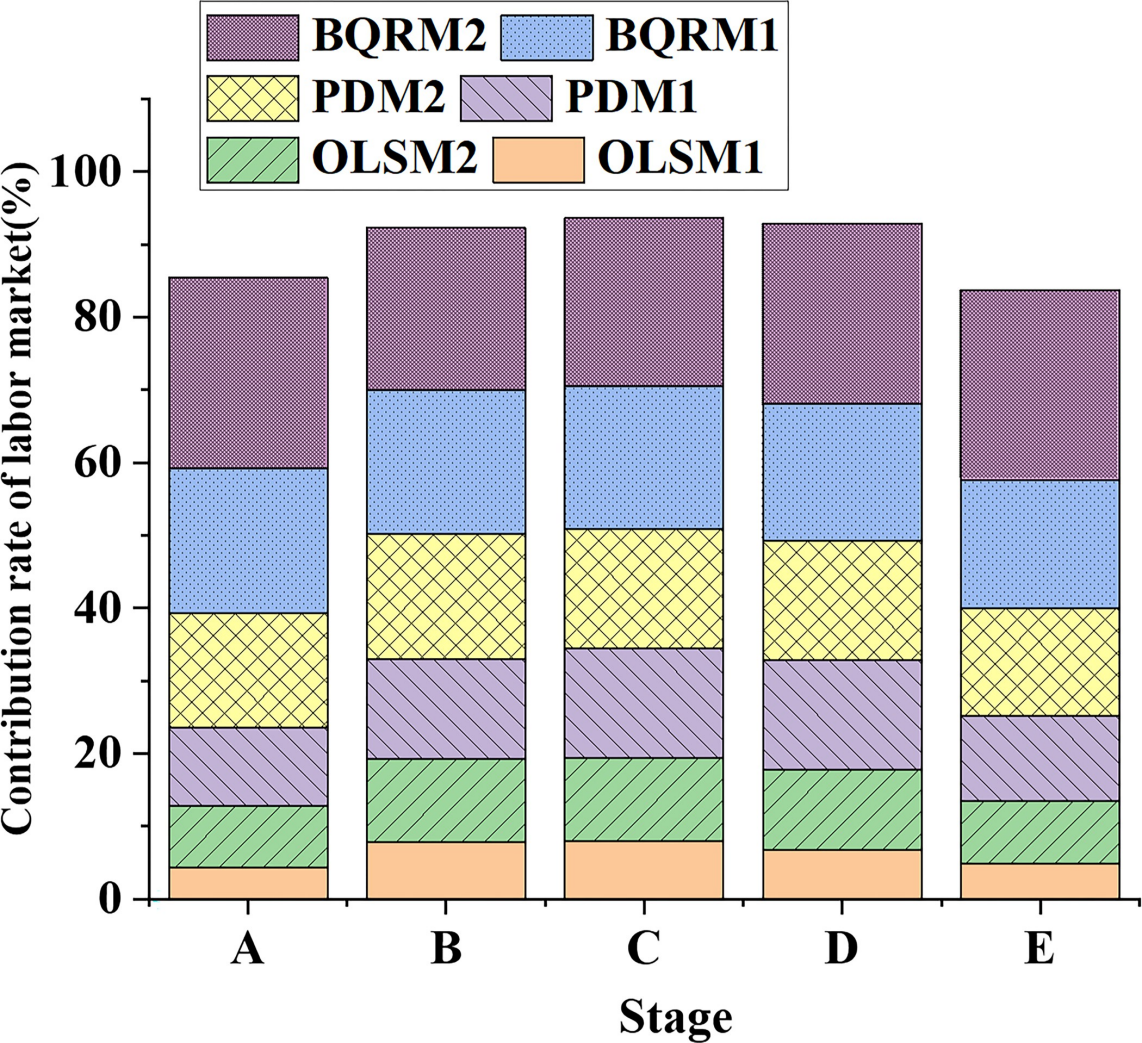
Data variation curves of labor market contribution rates in different industrial structure models at different time points.

The data variation trends of capital investment contribution rates in different industrial structure models at different time points are shown in Fig 5. The data variation trends of labor market contribution rates in different industrial structure models at different time points are shown in Fig 6.

Fig 5 compares and analyzes the contribution rates of capital investment and the labor market for different industrial structure models. Regarding capital investment, BQRM1 and BQRM2 consistently exhibit higher contribution rates, indicating that the BQRM has higher accuracy and precision in estimating the contribution rate of capital investment. Meanwhile, over the past 20 years, the contribution rates of capital investment for different industrial structure models have shown a gradual increase, further emphasizing the growing importance of capital investment to economic growth. In the labor market aspect, there are variations in the contribution rates of different models at different stages. BQRM1 and BQRM2 demonstrate notably high contribution rates in the later stages, showing a trend of gradual increase. These findings contribute to a more comprehensive understanding of how capital investment and the labor market impact economic growth in terms of modes and magnitudes.

In Fig 6, during the early stages A and B, OLSM1 and OLSM2 show relatively low labor market contribution rates, while PDM1 and PDM2 exhibit slightly higher contribution rates. However, in the later stages, C, D, and E, BQRM1, and BQRM2 show significantly higher labor market contribution rates compared to other models, and they also demonstrate a trend of gradual increase. Therefore, when analyzing the labor market contribution rates, it is important to consider the results of different models, particularly the more accurate and reliable estimates obtained through the BQRM.

### 4.3 Analysis of industrial structure adjustment and employment structure improvement performance

**Fig 7:**
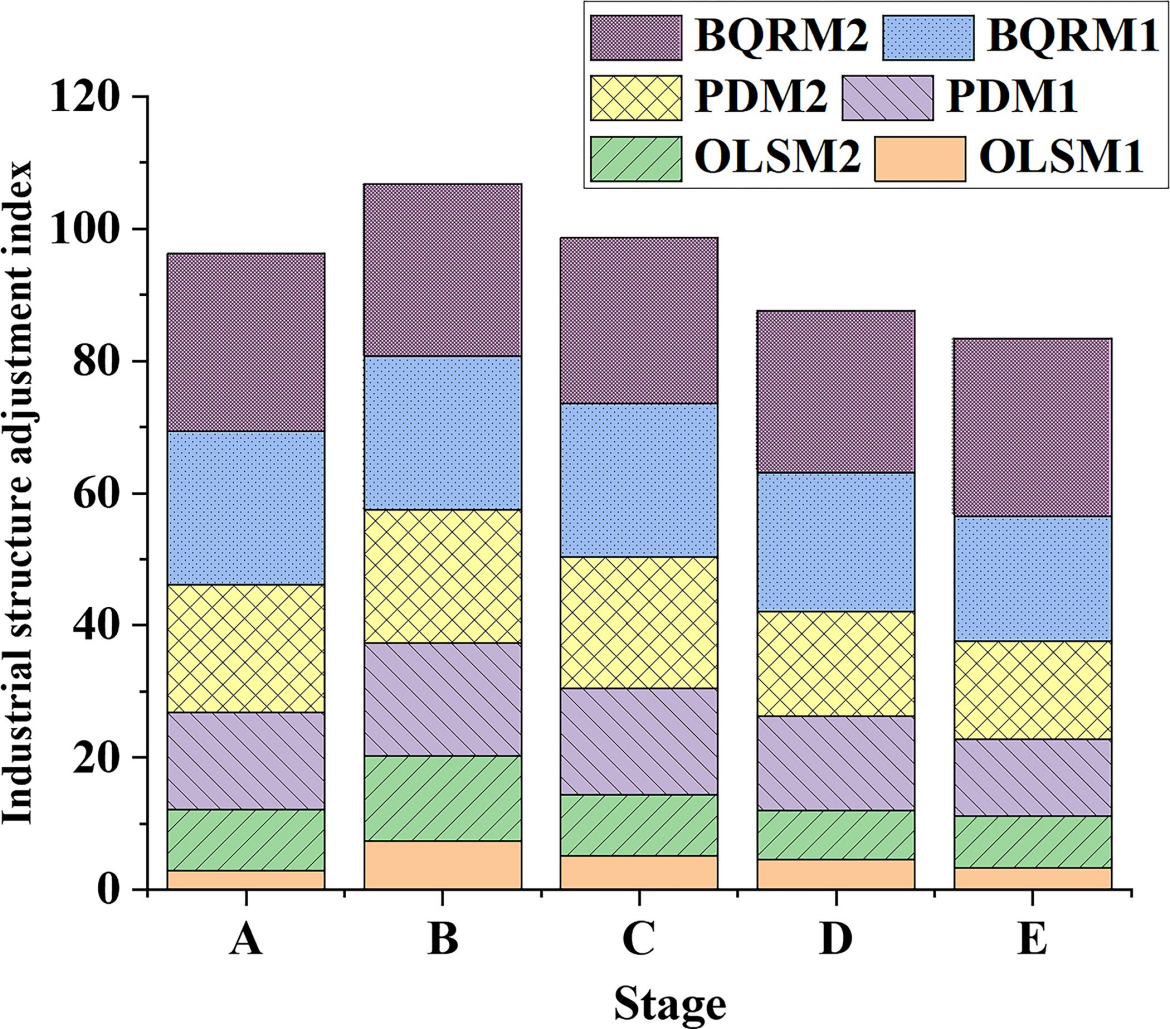
Data variation curves of industrial structure adjustment index in different industrial structure models at different time points.

**Fig 8:**
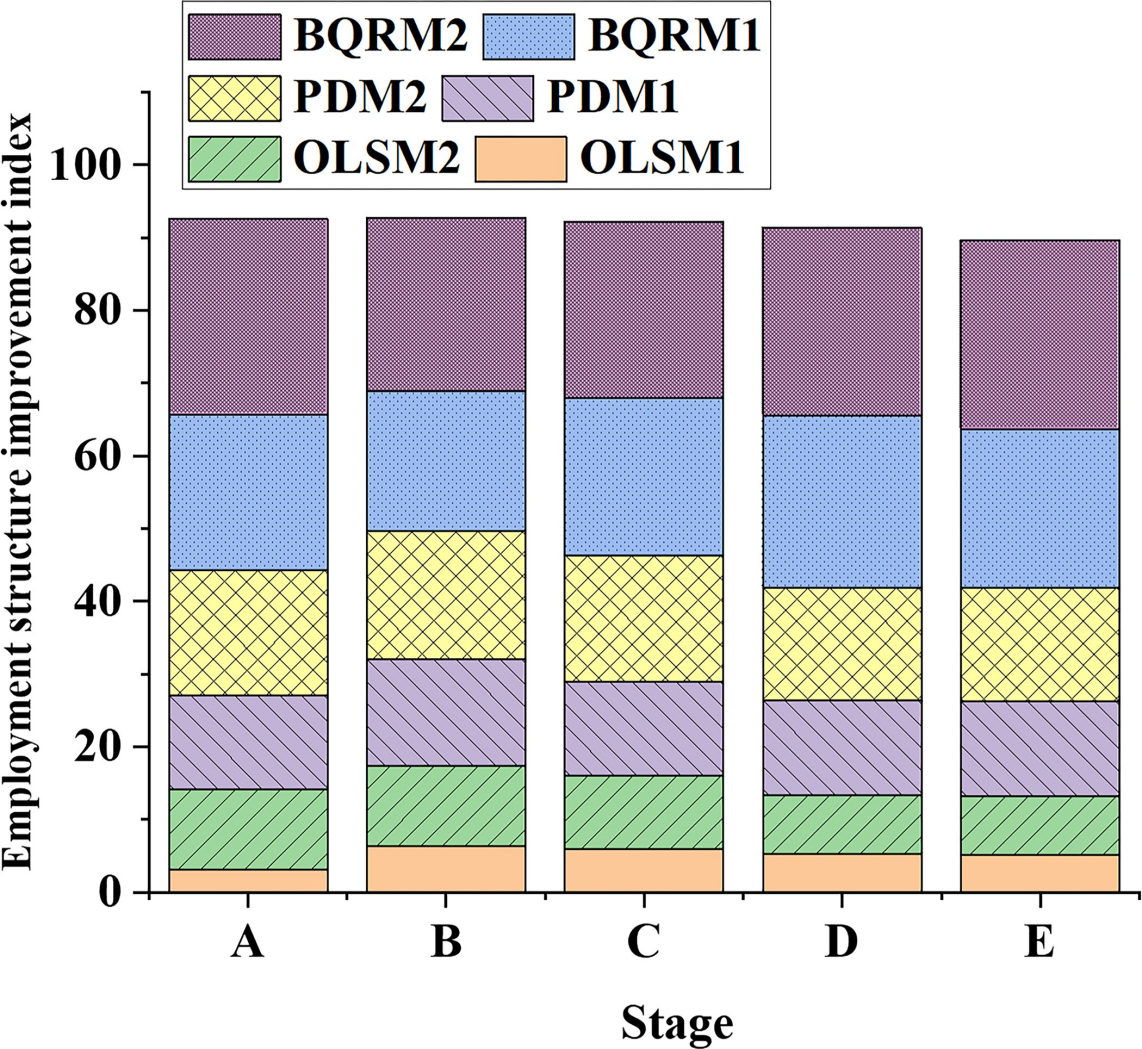
Data variation curves of employment structure improvement index in different industrial structure models at different time points.

In order to analyze the effects of industrial structure upgrading on employment structure improvement and the variations in human capital effects, data on the industrial structure adjustment index from different time points and different industrial structure models are collected and presented in Fig 7. Additionally, data on the employment structure improvement index from different time points and different industrial structure models are illustrated in Fig 8.

Fig 7 provides a detailed comparison of the impact of industrial structure adjustments on improving employment structure. It examines the performance of different industrial structure models at different stages and explores the mechanisms and reasons behind their performance. At different stages, the industrial structure adjustment indices of each model exhibit varying trends. BQRM1 and BQRM2 consistently show higher values across all stages, indicating the superiority of the BQRM in analyzing industrial structure adjustments.

In Fig 8, a consistent increasing trend is observed in the employment structure improvement for OLSM1 and OLSM2 models at various stages, while BQRM1 and BQRM2 consistently exhibit higher levels of employment structure improvement indices throughout the entire study period. These results provide crucial insights into the dynamic changes during the industrial structure adjustment process, emphasizing the contributions and advantages of different models in the analysis.

### 4.4 Results of data skewness, kurtosis, and Jarque-Bera test

In the study, basic statistical measures such as skewness, kurtosis, and the Jarque-Bera (JB) test are computed to offer a comprehensive data analysis and interpretation of results. Firstly, the experiment calculates the skewness and kurtosis of the data, helping to understand the shape and symmetry of the data distribution. Skewness measures the degree of skewness in the data distribution, while kurtosis measures the sharpness of the data distribution. Secondly, the Jarque-Bera (JB) test is conducted, which is a statistical test used to check whether the data follows a normal distribution. The JB test assesses the suitability and accuracy of the adopted statistical model by determining if the data conforms to the assumption of normal distribution. By computing these basic statistical measures, the experiment gains a more comprehensive understanding of the data’s characteristics and distribution, further enhancing the interpretation and understanding of the research results. The results of data skewness, kurtosis, and the Jarque-Bera test are presented in Table 7.

**Table 7:** Data skewness, kurtosis and results of Jarque-Bera test.

Statistical indicators	Value
Skewness	-0.24
Kurtosis	2.18
JB test statistic	4.62
JB test p-value	0.098

The conclusions from Table 7 indicate a skewness of -0.24, suggesting a slight leftward skewness in the data, but the skewness is close to 0, indicating relative symmetry in the data distribution. The kurtosis is 2.18, signifying a slightly sharper distribution compared to a normal distribution. The Jarque-Bera test statistic is 4.62, with a corresponding p-value of 0.098. As the p-value exceeds the significance level of 0.05, the experiment cannot reject the hypothesis that the data follows a normal distribution. Combining these results, it can be tentatively inferred that the data distribution is slightly skewed and sharp, but still generally adheres to the assumption of a normal distribution.

### 4.5 Discussion

To further validate the research results and strengthen the theoretical foundation of the relationship between human capital and economic growth, this study conducts an in-depth analysis of the crucial role of human capital in economic growth. Specifically, the experiment references the Solow model and endogenous growth theory, two theories that provide important perspectives for understanding the impact of human capital on economic growth. The Solow model attributes economic growth to capital accumulation, labor force growth, and technological progress, with a particular emphasis on the quality of labor, i.e., human capital, in driving economic growth. According to the Solow model, the enhancement of human capital promotes long-term economic growth by increasing the productivity of laborers. As education levels rise and skills are developed, workers can more effectively utilize existing technologies and even innovate, thereby propelling overall economic growth. Furthermore, endogenous growth theory overcomes some limitations of the Solow model, especially by highlighting the endogenous role of knowledge, technological progress, and innovation in economic growth. In this framework, human capital is not only a factor driving economic growth but also a core driving force. Endogenous growth theory posits that by enhancing education and skills, human capital can be strengthened, which, in turn, promotes technological innovation and the accumulation of knowledge. This process of knowledge accumulation not only directly stimulates economic growth but also accelerates growth by fostering the development and application of new technologies. Therefore, this study draws on the core viewpoints of the Solow model and endogenous growth theory to delve into how the improvement of human capital drives economic growth by enhancing labor productivity and promoting technological innovation. Such a theoretical framework not only deepens our understanding of the mechanisms through which human capital operates in economic development but also provides a solid theoretical foundation for the significant impact of human capital on economic growth.

Further discussion of these findings reveals some trends that contribute to a better understanding of the research outcomes and the addressing of research hypotheses. First, from the perspective of human capital, there are significant differences in the contribution rate of human capital at different time points among different industrial structure models. During high-speed growth phases, human capital’s contribution to economic growth is relatively low. However, during periods of industrial structural adjustment, this contribution rate significantly increases. As industrial structure evolves, the importance of human capital gradually increases. This conclusion aligns with the hypothesis, which posits that human capital plays a more substantial role during the process of industrial structural upgrading. Therefore, the results validate this hypothesis, and they are similar to the findings of Zhao et al. (2022) [62], who analyzed the role of the digital economy in industrial structural upgrading. Empirical results indicate that the development of the digital economy has a significant positive direct impact on industrial structural upgrading, as measured by the indicators of advancement and service orientation.

Second, concerning technological progress, the contribution rate of technological progress at different time points shows varying trends among different industrial structure models. This data indicates that each model has different effects on promoting technological progress at different stages. However, the overall trend shows that technological progress gradually enhances its role in industrial development. This suggests that technological innovation plays a crucial role in industrial structural upgrading, consistent with the hypothesis of this study. Regarding capital investment and the labor market, introducing the BQRMmethod can provide more accurate and precise estimates of the contribution rate of capital investment. Furthermore, the contribution rates of capital investment and the labor market for different models show an upward trend over time, indicating a gradual increase in their contributions to economic growth. This aligns with our hypothesis that these factors positively affect industrial structural upgrading. Conti et al. (2023) [63], based on Bayesian assessments combined with sign and narrative constraints and using Italian data, provide new reliable estimates of the impact of shocks to bank capital requirements on loan supply and macroeconomic activity, thus confirming the effectiveness of the BQRM.

Finally, concerning industrial structural adjustment and employment structure improvement, the BQRM exhibits higher precision in estimating the industrial structural adjustment index. Different models’ industrial structural adjustment indices show certain trends, especially in the later stages of industrial structural adjustment. The results indicate that BQRM has an advantage in analyzing the degree of industrial structural adjustment and the evolutionary patterns of different industries. Additionally, the increasing trend in the employment structure improvement index suggests that changes in industrial structure have a positive impact on employment structure improvement. This may be due to changes in labor market demand during industrial structural adjustment, resulting from changes in industrial structure. This leads to an increased demand for higher-skilled and more flexible labor, emphasizing the importance of education and vocational training. This also implies more investment in human capital, thus increasing the contribution of human capital to economic growth. Industrial structural upgrading is typically accompanied by technological innovation and transformation. Higher levels of human capital can better cope with the application and innovation of new technologies, thereby promoting the role of technological progress in the industry. Simultaneously, higher levels of human capital mean better skills matching, which can enhance labor market efficiency, reduce structural unemployment rates, and further promote economic growth.

In relation to the impact of human capital on economic development, Duan et al. (2022) explored the relationships among human capital, economic freedom, governance performance, and economic growth. After multiple mixed methods tests, the study found that the relationship between human capital and economic growth exhibits a nonlinear, inverted U-shaped pattern [64]. Moreover, within a certain timeframe, human capital positively influences economic growth, and governance performance positively moderates the impact of human capital on economic growth in BRICS countries. This aligns with this study’s findings, indicating that human capital’s influence on economic growth during different stages of industrial structural upgrading exhibits a stage-specific nonlinear trend. Additionally, Duan et al.’s research highlighted the positive moderating effect of governance performance on the impact of human capital on economic growth in BRICS countries, resonating with the policy recommendations mentioned in this study regarding government support for training and skill development. Wirajing et al. (2023) suggested that economic growth in Africa is positively influenced by the development of human capital [65]. Wirajing et al.’research supported the conclusions of this study, emphasizing stage-specific variations in the contribution of human capital to economic growth during different stages of industrial structural upgrading. Mehmood (2022) examined the impact of human capital, financial development, and institutional quality on the ecological footprint of 11 country groups [66]. The study found that institutional quality and human capital increased environmental quality by 0.07% and 0.01%, respectively. This is relevant to the policy recommendations in this study, emphasizing the importance of government support for training and skill development to promote sustainable economic growth. Xia et al. (2022) focused on the role of human capital and remittances in economic growth, discovering a positive and significant association between remittances and human capital development [67].

In the context of the impact of human capital on economic development during the process of industrial structure upgrading, Lazaroiu et al.’s (2023) research on the relationship between AI-driven economic growth and green total factor productivity provided important insights into the effects on employment, business value, and customer engagement [68]. Their study offered a valuable set of research findings and theoretical contributions. Ciobanu et al. (2019) emphasized that although public employees were mainly driven by intrinsic motivation and dedication to public values and interests, a supportive work environment also positively influenced their performance [69]. Their research underscored the importance of public values for the performance of public sector employees and how management practices could enhance the work motivation and performance of public employees. Cramarenco et al. (2023) presented evidence highlighting the difficulties arising from skill upgrading or retraining efforts in response to technological changes [70]. They pointed out that efforts to address skill mismatches affected employees’ well-being during challenging pandemic periods. The scholars’ research provided crucial theoretical and practical references for a deeper understanding of the impact of AI on employees’ personal and professional lives, offering insights for organizational managers to better cope with ongoing technological changes and challenges. Additionally, this study focuses on the role of human capital in economic development and employs BQRM to more accurately comprehend the influence of human capital on economic growth at different stages. Meanwhile, He et al. (2023), utilizing the Tapio decoupling model, examined the past reduction status of the agricultural grey water footprint (AGWF) in the context of economic development. They categorized the two-dimensional decoupling status between AGWF and agricultural GDP, aiming to guide the implementation of zonal management and proposed a comprehensive analytical framework for differentiated zonal management of regional agricultural water pollution disparities [71]. In addressing the importance of cooperation in water pollution management across jurisdictional river basins, Yuan et al. (2024) established a dynamic differential game model for cross-jurisdictional river basin pollution management. The model involved upstream and downstream governments as well as sewage enterprises. The researchers computed the strategic equilibrium solution for multiple stakeholders, analyzed the variations in strategic choices among different stakeholders under cost-sharing and non-cost-sharing scenarios, and simulated stakeholders’ strategic choices in various scenarios. The study made recommendations from the perspectives of cooperative strategies, reward-punishment mechanisms, and the application of advanced technological equipment to enhance collaboration in managing water pollution across jurisdictional river basins [72]. Firstly, this study exhibits distinct differences from He et al.’s research. While He et al. focused on reducing agricultural water pollution, this study concentrates on the impact of human capital at different stages of economic development. These two research directions have different subjects and focal points. Secondly, compared to Yuan et al.’s study, this study emphasizes the role of human capital in economic development rather than cross-jurisdictional river basin water pollution management. The two studies differ in research objectives, methods, and conclusions. The former delves into the role of human capital through BQRM, while the latter investigates cooperation in water pollution management using a dynamic differential game model. Despite the differences in research topics, this study addresses a research gap by examining the influence of human capital on economic development during the industrial structure upgrading process, providing policymakers with more targeted and effective recommendations. Together with the studies by He et al. and Yuan et al., this study showcases the diversity of in-depth research in different domains, collectively offering valuable academic references for sustainable development. This highlights that comprehensive policy formulation in economic, agricultural, and environmental fields can benefit from multi-faceted in-depth research.

In terms of policy formulation, the study recommends providing additional incentive measures to encourage immigrants to send more remittances into the economy, ultimately supporting sustainable economic growth. Furthermore, an efficient and effective financial sector can ensure optimal utilization of the economy through channels of capital formation; therefore, nations must focus on establishing efficient financial intermediaries. This aligns with the promotion role of human capital in the industrial structural upgrading process discussed in this study. Both emphasize the consideration of strengthening support for human capital development in policy formulation to drive economic growth. In summary, these scholars’ research provides robust support and context for this study. Through dialogue with these relevant studies, the results of this study receive strong theoretical and empirical support while also offering substantial recommendations for policy formulation. Together, these studies underscore the critical role of human capital in economic development and the complex impact on economic growth in different contexts.

In summary, the research results largely validate the hypotheses. During industrial structural upgrading, factors such as human capital, technological progress, capital investment, and the labor market gradually increase their contributions to economic growth and industrial development. Furthermore, the study highlights the superiority of the BQRM in analyzing industrial structure models, providing more accurate and reliable estimation results. These findings are of significant importance for policy formulation and the development of industrial upgrading strategies.

## 5. Conclusion

This study employed OLSM, PDM, and BQRM to thoroughly analyze the mechanisms of different industrial structure models in economic growth. The analysis of the contribution rate of human capital revealed differences among OLSM, PDM, and BQRM in enhancing the contribution of human capital to economic growth at different stages of industrial structural adjustment. Particularly noteworthy is that the BQRMl exhibited a higher contribution rate of human capital during the adjustment period, suggesting its greater accuracy in explaining the impact of human capital on economic growth. Regarding technological progress, the experiment analyzed the contribution rates of different models at different time points. The study found that overall, the contribution rates of technological progress for each model showed a gradual increase, reflecting the progressive strengthening of the role of technological progress in economic growth. In the early stages, the contribution rates of technological progress for each model were relatively low and gradually increased over time. This might be because technological innovation requires a certain time for accumulation and dissemination to fully unleash its potential. In subsequent stages, the contribution rates of technological progress for each model remained relatively stable. In terms of capital investment, comparing the performance of different models in capital investment contribution rates showed that the BQRM consistently exhibited higher capital investment contribution rates throughout the study period. Especially, the BQRM more accurately estimated the contribution of capital investment to economic growth compared to other models. This implies that in formulating industrial policies and attracting investments, the information provided by the BQRM has more practical application guidance. In the labor market aspect, this study compared the contribution rates of different models at different time points. The results indicated that the BQRM exhibited higher labor market contribution rates in later stages, emphasizing its advantages in explaining improvements in employment structure. Therefore, in formulating labor market policies, more consideration should be given to the analytical results of the BQRM. Regarding literature contribution, the research filled some gaps in existing studies regarding the mechanisms of the impact of industrial structural adjustment on economic growth. The experiment compared the consistency and differences between the results of this study and relevant literature, pointing out the innovations of this study in both theory and empirical aspects. This provides the academic community with deeper space for thought and discussion. Finally, based on the research findings, this study proposed a series of policy recommendations. For example, in terms of human capital, the government can promote positive contributions of human capital to economic growth by strengthening the education system and improving workers’ skill levels. For technological progress, encouraging research institutions and enterprises to increase research and development investment can facilitate the rapid dissemination of technological innovation. Regarding capital investment, the government can offer more incentive measures to attract more investments into critical areas. In the labor market aspect, it is suggested to formulate more flexible and market-oriented employment policies to promote the healthy development of the labor market. These policy recommendations aim to guide relevant stakeholders in making wiser decisions in practice to drive comprehensive and sustainable economic growth.

Although this study has made important findings, there are still some limitations. Firstly, this study focuses on provincial-level administrative regions in China, and future research could consider expanding the sample to cover more countries and regions. Furthermore, the constructed models can still be further improved to capture the complex relationships among various factors more accurately during the process of industrial structural upgrading. Future research could also explore the impact of industrial, innovation, and labor market policies on industrial structure. This study provides new insights into the factors influencing industrial structural upgrading and offers important recommendations for policymakers. However, many future research directions still need further exploration to deepen our understanding of this critical topic.

## Supporting information

S1 Data (ZIP)
