# Correction: Analysis of human capital effects introducing Bayesian quantile regression in the process of industrial structural upgrading

**DOI:** 10.1371/journal.pone.0322560

**Published:** 2025-04-08

**Authors:** Xinbo Wang, Shaodong Shi

The authors are listed out of order. Please view the correct author order, affiliation, and citation here: Xinbo Wang, Shaodong Shi,

1 Lomonosov Moscow State University, Moscow, Russian Federation

Wang X, Shi S (2024) Analysis of human capital effects introducing Bayesian quantile regression in the process of industrial structural upgrading. PLoS ONE 19(7): e0304730. https://doi.org/10.1371/journal.pone.0304730
